# Inter-Limb Muscle Synergies and Kinematic Analysis of Hands-and-Knees Crawling in Typically Developing Infants and Infants With Developmental Delay

**DOI:** 10.3389/fneur.2018.00869

**Published:** 2018-10-16

**Authors:** Qi L. Xiong, Xiao Y. Wu, Jun Yao, Theresa Sukal-Moulton, Nong Xiao, Lin Chen, Xiao L. Zheng, Yuan Liu, Wen S. Hou

**Affiliations:** ^1^Key Laboratory for Biorheological Science and Technology of Ministry of Education, Chongqing University, Chongqing, China; ^2^Chongqing Medical Electronic Engineering Technology Research Center, Chongqing University, Chongqing, China; ^3^Collaborative Innovation Center for Brain Science, Chongqing University, Chongqing, China; ^4^Department of Physical Therapy and Human Movement Sciences, Northwestern University, Chicago, IL, United States; ^5^Department of Rehabilitation, Children's Hospital of Chongqing Medical University, Chongqing, China

**Keywords:** infant crawling, EMG, muscle synergy, kinematics, motor developmental delay

## Abstract

Hands-and-knees-crawling is an important motor developmental milestone and a unique window into the development of central nervous system (CNS). Mobility during crawling is regularly used in clinical assessments to identify delays in motor development. However, possible contribution from CNS impairments to motor development delay is still unknown. The aim of this study was to quantify and compare inter-limb muscle synergy and kinematics during crawling among infants at a similar developmental age, however, clinically determined to be typically developing (TD, *N* = 20) infants, infants at risk of developmental delay (ARDD, *N* = 33), or infants with confirmed developmental delay (CDD, *N* = 13). We hypothesized that even though all of the groups are at a similar developmental age, there would be differences in kinematic measures during crawling, and such differences would be associated with CNS impairment as measured by electromyography (EMG) features. Surface EMG of eight arm and leg muscles and the corresponding joint kinematic data were collected while participants crawled on hands and knees at their self-selected velocity. Temporal-spatial parameters and normalized Jerk-Cost (JC) function (i.e., smoothness of movement) were computed from the measured kinematics. The inter-limb muscle synergy and the number of co-activating muscles per synergy were measured using EMGs. We found that the infants with CDD demonstrated higher normalized JC values (less movement smoothness), fewer muscle synergies, and more co-activating muscles per synergy, compared to infants with TD (*p* < 0.05) and ARDD (*p* < 0.05). Furthermore, the normalized JC values were correlated (*p* < 0.05) with the number of co-activation muscles per synergy. Our results suggest a constrained neuromuscular control strategy due to neurological injury in infants with CDD, and such constrain may contribute to the reduced movement smoothness in infant crawling.

## Introduction

Mobility during hands-and-knees crawling is regularly used in clinical assessments to benchmark delay in motor development because it is an early example of skillful gross motor ability. Clinically, a delay can be quantified relative to typical achievement of gross motor milestones (1), but the extent to which such delay is related to CNS impairment cannot be ascertained. Kinetic and kinematic measures during 4-beat crawling can enrich infant assessment and provide a non-invasive window to CNS function.

Previous studies have measured kinetics and kinematics separately in infants crawling. Early studies used film recording to investigate the movement pattern of limbs while crawling from a small sample size of infants (*N* = 7) (2), while more recent studies used 3D motion capture to examine inter-limb coordination patterns during crawling. The typical pattern of crawling is with diagonal limbs tending to move together and ipsilateral limbs alternating during crawling on hands and knees (3, 4). Quantitative data concerning muscle activities in human infant crawling is sparse. It has been briefly described as triceps brachii activation throughout the stance phase of the arm during crawling, with quadriceps femoris activated during swing phase of the leg (4). Our previous work demonstrated that muscle co-activations of lower extremities during crawling is correlated to their motor skill development (5), but it only quantified the coordination between antagonist muscles of a single limb, which provides little information about the inter-limb coordination across arm and leg muscles during hands-and-knees crawling.

Muscle synergy analysis is a valid tool to explore the coordination across multiple muscles during locomotion (6), and reflects the CNS control for locomotion as a linear combination of several muscle activation patterns in order to complete functional tasks. Crawling is a self-motivated rhythmic locomotion that involves controlled inter-limb muscle coordination for movement. Thus, quantifying inter-limb muscle synergy during crawling has the potential to explore the underlying factors related to movement abnormalities related to the changes/impairments of the CNS.

Muscle synergy extraction based on surface electromyography (sEMGs) and non-negative matric factorization (NMF) algorithm has been used to explore muscle coordination during locomotion in neurotypical populations as well as a number of pathologic conditions, such as stroke (6), spinal cord injury (7), or cerebral palsy (8). For instance, Dominici et al. concluded that two basic muscle synergies are retained through infant development, and are augmented by new synergies during the development of independent walking (9). Steele et al. found that individuals with CP (age range 3.9–70 years) demonstrated fewer synergies during gait compared with unimpaired individuals (8), similar to the constrained muscle control found in adults following stroke (6, 10). Muscle synergy analysis has also been used to quantify the kinetic feature during hand-and-knee crawling. Chen's study (11) extracted two alternative intra-limb muscle synergies during crawling in healthy adults, with one related to the stance phase and the other related to the swing phase (11). However, muscle synergy analysis during infant crawling has not been systematically investigated either in typical development or neurological disorders.

In order to fill the gap, we simultaneously measured kinetic (i.e., EMG) and kinematic features during crawling with typically developing infants and infants with different risks or severities of developmental delay. Because smoothness and well-coordinated movement are typical features of well-developed human motor behavior (12), we expected that kinematic output, such as the smoothness of movement, would be altered in infants with developmental delay. At the same time, we hypothesized that CNS control in infants with developmental delay is also impaired, and would be manifested in the metrics of muscle synergy. Finally, we hypothesized that CNS development/impairment would be associated with the movement smoothness.

## Methods

### Participants

We recruited 47 atypically developing infants (age range 8–43 months, 14.21 ± 6.91 months; female: *N* = 20, male: *N* = 27) from the Department of Rehabilitation Center, Children's Hospital of Chongqing Medical University. Infants visited the hospital to follow up for the risk of developmental delay (13) due to: (1) premature delivery (gestational age <37 weeks); (2) low birth weight (<2,499 grams), regardless of gestational age; or (3) lack of oxygen to the brain during birth. The age of one infant was 43 months, which is far from the distribution of other infants' age and therefore was excluded as an outlier. The remaining 46 infants (age range 8–32 months, 12.78 ± 4.87 months) were included for data analysis in this study. In addition, 20 developmental-age-matched healthy infants (age range 8–15 months, 10.95 ± 2.25 months; female: *N* = 9, male: *N* = 11) were recruited from local child health clinics as “typical development (TD)” controls. They were all full-term with normal birth weight, and no diagnosed health conditions per parent report. All infants were studied at the Department of Rehabilitation Center, Children's Hospital of Chongqing Medical University. The experiments were performed with informed, written consent of the parents or guardians of the infants, and the procedures were approved by the ethics committee of Children's Hospital of Chongqing Medical University (approval number: 065/2011). Partial results (i.e., crawling velocity, cadence, stance phase time) from the 20 TD infants have been published before (5).

### Clinical assessment

For all of the participants, the Gross Motor Function Measure (GMFM-88) and Gesell Developmental Scale were assessed by specialist physicians. GMFM-88 measures gross motor function, including lying and rolling, crawling and kneeling, sitting, standing, walking activities. Each function is scaled in the range of 0–100 (1). Gesell Developmental Scale is a set of developmental metrics, which assesses the ages and stages of development in young infants (1).

For the infants who were atypically developing, developmental age (see the column 2 in Table 1) was assessed by the gross motor development part of Gesell Scale, and compared to their biological age to calculate delayed age (in months) for each of the infants (see the column 1 in Table 1). Infants with a developmental delay of ≤3 months were classified as at risk of developmental delay and those with a delay larger than 3 months as having confirmed developmental delay. This resulted in 13 infants (age: 20.15 ± 5.85 months, delayed age: 8.65 ± 4.47 months) with confirmed developmental delayed (CDD), and 33 infants (age: 11 ± 2.29 months, delayed age: 0.3 ± 0.63 months) who were at risk of developmental delay (ARDD). Although the biological age of CDD group is larger than that for TD and ARDD groups (*F* = 41.50, *p* < 0.01), the developmental age of all the groups are similar (*F* = 0.072, *p* = 0.790), demonstrating clinically comparable level of motor skills in all the 3 groups. Demographic information for all participants is summarized in Table 1.

**Table 1:** Participant demographic information.

	**Biological age (months)**	Delayed age^*^(months)	**Scale score of five dimensions assessed by gmfm-88 (%)**					**Number of strides for analysis**
			**Lying**	**Crawling & kneeling**	**Sitting**	**Standing**	**Walking**	
TD (*N* = 20)	10.95 ± 2.25	0.30 ± 0.73	90.35 ± 4.12	50.90 ± 4.48	83.05 ± 5.44	28.3 ± 17.77	11.65 ± 8.09	10.35 ± 2.70
ARDD (*N* = 33)	11 ± 2.29	0.30 ± 0.63	90.63 ± 5.06	50.48 ± 7.38	83.72 ± 4.70	24.57 ± 17.61	9.42 ± 9.69	6.54 ± 3.33
CDD (*N* = 13)	20.15 ± 5.85	8.65 ± 4.47	91.23 ± 2.71	57 ± 10.16	83.38 ± 10.37	30 ± 24.63	15.84 ± 14.12	9.07 ± 2.49

* *Determined by Gesell Developmental Scale*.

### Protocol

Infants first became acquainted with the laboratory by spending time on a floor crawling mat (size 360 × 120 cm). Next, they were encouraged to crawl from one end to the other in response to toys or mother's calling. After training, infants wore only diapers. A motion capture system (Raptor-E, Motion Analysis Corporation, USA) was used to record kinematic movement of infants at 100 frames/s with six high-speed digital cameras. Fourteen reflective markers were taped over the shoulder (lateral to the acromion), elbow (lateral epicondyle), wrist (ulnar styloid process), hip (posterior superior iliac spine), knee (lateral joint line), ankle (lateral malleolus), and trunk (scapula).

**Figure 1:**
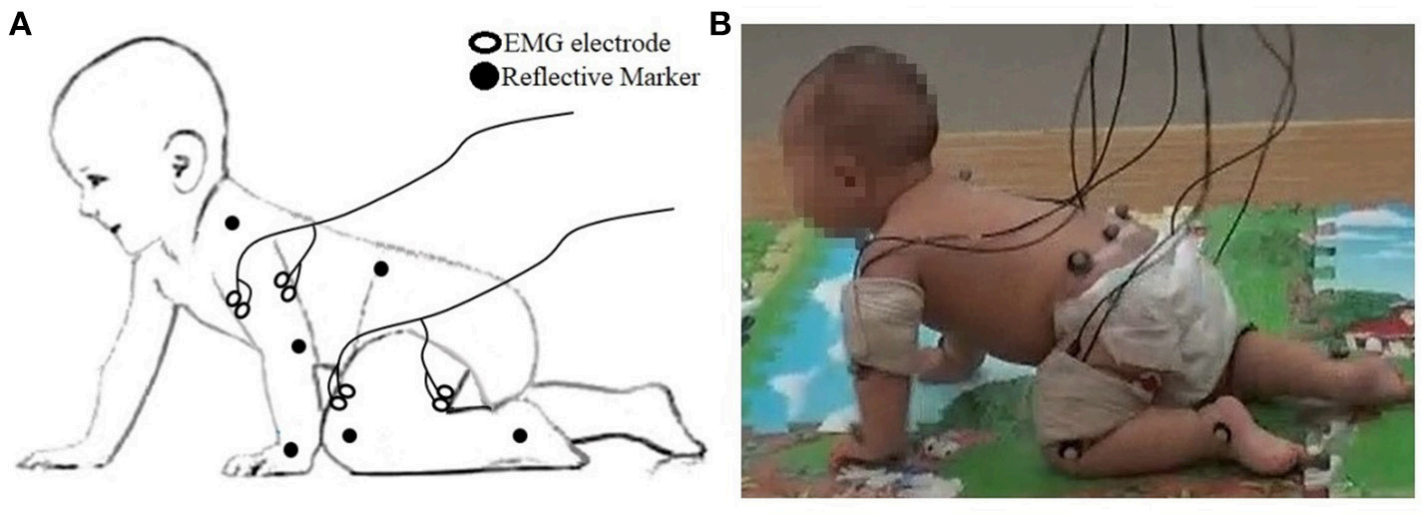
**(A)** The placement of the EMG electrode and reflective markers; **(B)** Snapshot of data collection.

Simultaneously, a surface EMG system (ME6000, Mega Electronics Ltd, Finland, bandwidth of 15–500 Hz) with pre-amplified EMG sensor units was used to measure sEMG from bilateral arm and leg muscles, including: left and right triceps brachii (LTB, RTB) and biceps brachii (LBB and RBB), quadriceps femoris (LQF and RQF) and hamstring (LHS and RHS) (see Figure 1) by differential electrodes. All of the sEMG was sampled at 1 kHz and synchronized with kinematic data recording by a TTL pulse. In addition, movements of participants were videotaped.

A valid trial was defined as straight crawling without stop or deviation, for at least three complete, consecutive strides. In each of the participants, the number of valid trials collected varied from 2 to 16 (on average 6.80 ± 3.60), depending on the cooperation of the infant.

### Data analysis

The first and last strides of each valid trial were excluded from the following data analysis.

#### Kinematic analysis

##### Temporal-spatial parameters

Missing raw kinematic data was constructed using cubic spline interpolation. Then they were low-pass filtered (6 Hz) with a zero lag 4th-order Butterworth filter to remove high frequency noise. In the current study, we defined the left wrist as the start of the crawl cycle, similar to the heel strike in gait analysis). The temporal-spatial crawling parameters were accordingly calculated from the 3D trajectories of the left wrist, including velocity, cadence, and stance phase time (normalized to crawling cycle, SPT), using the methods previously reported (5).

##### Movement smoothness

Movement smoothness was quantified by evaluating the endpoint jerk-cost (JC) at the left wrist, defined as:

(1) $$\begin{array}{r} \text{JC}=\overset{T}{\underset{0}{\int }}{\left(\frac{d^{3}s}{dt^{3}}\right)}^{2}dt \end{array}$$

where T is the total duration of a crawling cycle, and s is the position vector of the limb segment. JC measures the change between acceleration and deceleration during movement. A smaller JC value reflects fewer such switches and thus indicates a smoother movement (14).

For each crawling cycle of each subject, the endpoint JC of the wrist marker was calculated in anterior-posterior (AP) direction (JC_*x*_), medial-lateral (ML) direction (*JC*_*y*_), and vertical (VT) direction (*JC*_*z*_) using Equation (4). To account for variations in crawling velocity and to standardize results, all JC values were normalized by the total duration of each crawling cycle, T. Per subject, the normalized JC were then averaged across all valid crawling cycles.

#### EMG analysis

##### EMG preprocessing

The sEMG signals were band-pass filtered (10–400 Hz) using a 4th-order, zero-phase Butterworth digital filter and a 50 Hz digital notch filter for reducing the power interference. The filtered sEMG signals were then divided into segments according to the initiation of each crawling cycle. Segments of filtered sEMG signals were subsequently demeaned, rectified, and low-pass filtered with a zero lag 4th-order low-pass (9 Hz) to extract envelope. The envelope was then normalized to its peak value during each trial, then resampled from 0~100% of the crawling cycle at the 1% step increase. Finally, the normalized envelopes of per participant and per muscle were averaged cross all valid cycles. The averaged envelopes composed the EMG data matrix (8 × 101) for an individual subject during crawling.

##### Non-negative matrix factorization

A non-negative matrix factorization was applied to each EMG data matrix to extract muscle synergies. This method decomposed the measured EMG data matrices (M) into two components, spatial structure (W, termed the muscle synergies) and temporal structure (C, or relative activation of those synergies), as expressed by the following equation:

$$\begin{array}{l} M^{m\times t}=W^{m\times n}C^{n\times t}+\varepsilon \end{array}$$

**Figure 2:**
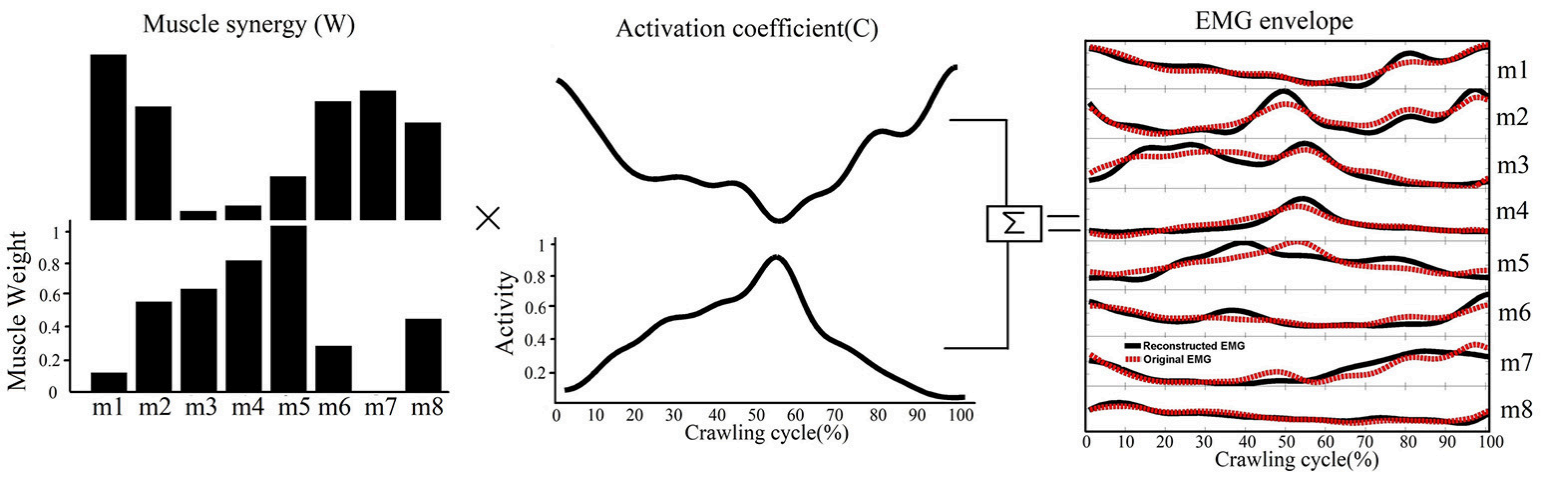
A schematic illustrating how muscle synergies are linearly combined to generate muscle patterns recorded as EMG signals. Each of the two muscle synergies (W) shown is represented as an activation weight across muscles (i.e., m1–m8) and activated through multiplication by a time-dependent coefficient(C). The EMG envelopes resulting from the activations of individual synergies are then summed together (black lines) to reconstruct the recorded EMG (red dashed line).

In this equation, W is an *m*×*n* matrix where m is the number of muscles (in this study *m* = 8) and n is the number of muscle synergies. C is an *n*×*t* matrix where t is number of time points (101 across the normalized crawling cycle in this study). ε represents the error between the measured EMG data (M) and the reconstructed EMG from W and C. Thus, each column of W represents the relative weighting of muscles in each synergy, and each row of C represents the activation level of each synergy over the gait cycle. Non-negative matrix factorization was repeated within an iterative optimization, which minimized the sum of squared error between the activations calculated by W × C and the measured EMG data (15). A typical decomposition result is shown in Figure 2.

##### Determining the number of muscle synergies

We made no a priori assumptions regarding the number of synergies (s) that would be needed to adequately reconstruct the original EMG. The goodness of fit of the data reconstruction was quantified by the variance accounted for (VAF, ranging 0–1), defined as *VAF* = 1 − ||ε||^2^/||*M*||^2^ (6, 16, 17). This is a similarity metric that is similar to Pearson correlation coefficient (*r*^2^). However, VAF is a more stringent criterion than *r*^2^ because it evaluates both shape and magnitude of the measured and reconstructed curves (17).

**Figure 3:**
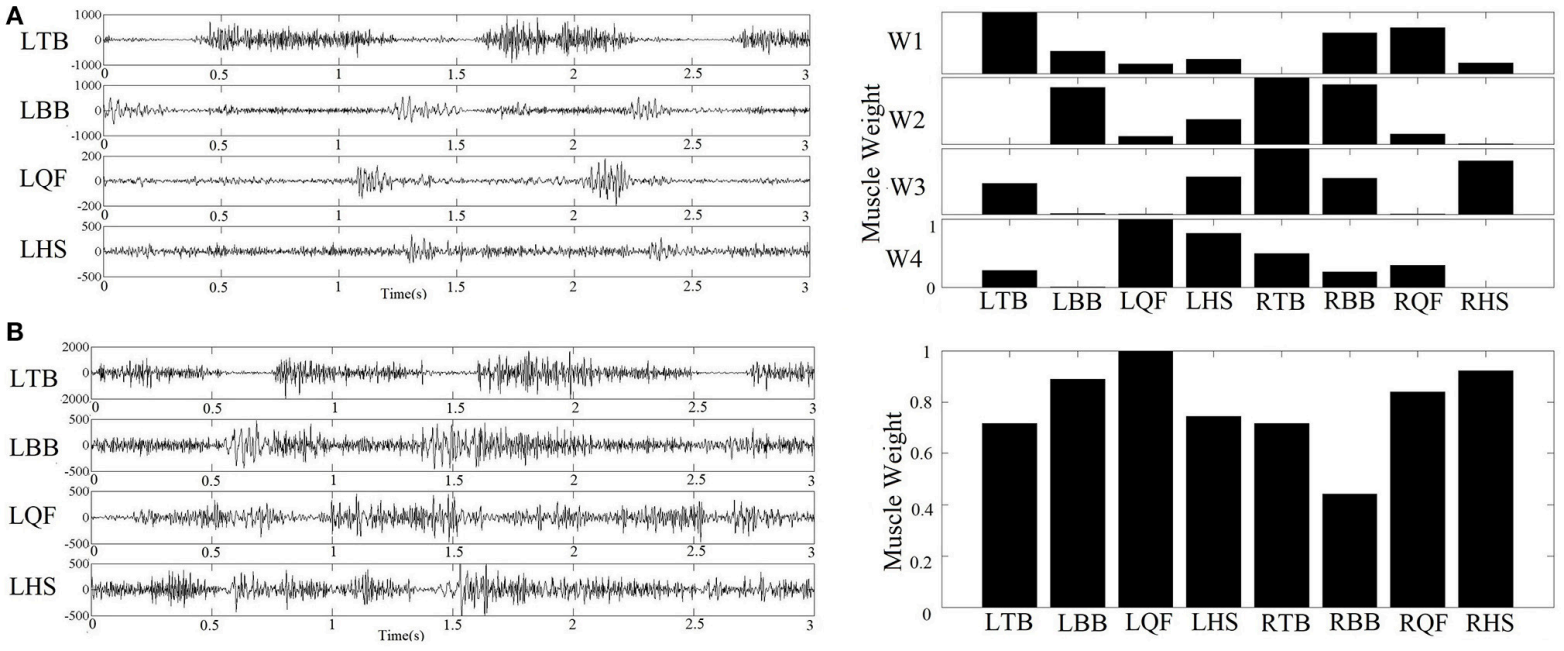
**(A)** Example of raw sEMG collected from an infant with typical development (left figure) and the corresponding four muscle synergy identified by NMF algorithm (right figure); **(B)** Example of raw sEMG collected from an infant with confirmed development delay (left figure) and the corresponding one muscle synergy extracted by NMF algorithm (right figure).

For each subject, we determined the least number of muscle synergies that satisfied the following 2 criteria: (1) the overall reconstructed EMGs accounted for at least 90% of the variance across all muscles (VAF>90%); and (2) each reconstructed EMGs accounted for >75% VAF of the measurement from the corresponding single muscle. These criteria are considered conservative to ensure goodness of reconstruction (6). An example of raw EMG signals and the corresponding muscle synergy was shown in Figure 3.

##### Quantifying the structure of muscle synergy

As an indicator of selective control and coordination, the number of co-activating muscles contributing to a single muscle synergy was calculated. Specifically, muscles in a synergy were defined as active if their normalized weight values exceeded 0.3 (18). Therefore, for each muscle synergy, the number of co-activating muscles varied from 8 (i.e., all the recorded muscles co-activated) to 1 (i.e., no co-activations). The number of co-activating muscles per synergy was calculated for each subject.

### Statistical analysis

Group difference in the number of muscle synergies, the number of co-activating muscles per synergy, and temporal-spatial parameters were compared using one-way ANOVA (factor of group) with *post-hoc* Bonferroni corrections for multiple comparisons.

In addition, a 2-way repeated measures ANOVA (within-subjects factor of directions (AP, ML, and VT), and group) was used for the dependent variable of the normalized JC value. A Bonferroni corrected *post-hoc* test was used if there was a significant effect.

Spearman rank correlation tests were performed for correlating kinetic indices (the number of co-activating muscles per synergy) and kinematics (normalized JC values). Significance level was set at *p* < 0.05. All analyses were performed using the statistical software package SPSS18.0. The results that showed a significant effect were marked with an asterisk in all figures.

## Results

### Comparison of the temporal-spatial parameters and normalized JC values

A one-way ANOVA found no significant effect of group for the temporal-spatial parameters of velocity (*F* = 0.445, *p* = 0.643), cadence (*F* = 0.289, *p* = 0.750), or stance phase time (*F* = 0.716, *p* = 0.493).

**Figure 4:**
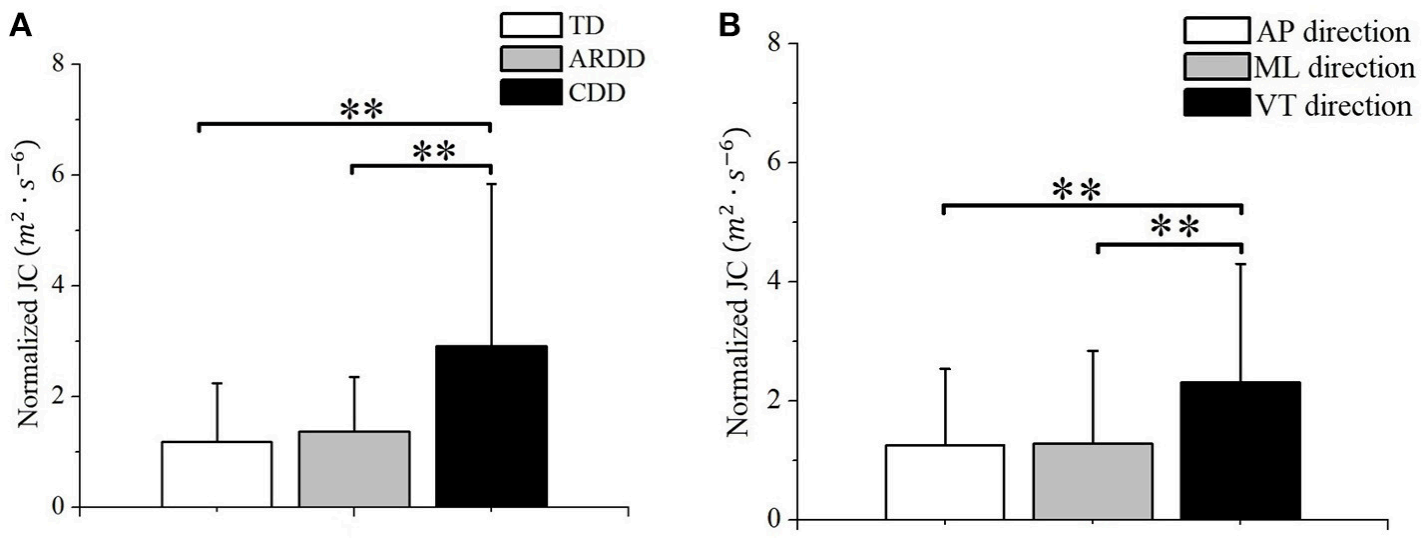
**(A)** Group difference between typical developing infants (TD), infants at risk of developmental delay, and infants with confirmed developmental delay (CDD) in the movement smoothness quantified by the averaged JC value across directions; **(B)** Direction difference between anterior-posterior (AP) direction, medial-lateral (ML) direction, and vertical (VT) direction in the movement smoothness quantified by the averaged JC value across groups. **indicates *p* < 0.01.

The 2-way repeated measures ANOVA showed a significant effect of group (*F* = 7.591, *p* < 0.01, observed power = 0.936) and direction (*F* = 34.301, *p* < 0.01, observed power = 1) on the normalized JC value. No significant interaction between these 2 factors was found (*F* = 1.24, *p* = 0.297). *Post-hoc* test using Bonferroni corrections revealed higher normalized JC values in the CDD group (averaged across AP, ML, and VT directions) compared to TD (*p* < 0.01) and ARDD (*p* < 0.01) groups (shown in Figure 4A). Further *post-hoc* testing showed higher normalized JC values (averaged across TD, ARDD, and CDD groups) in the VT direction compared to AP (*p* < 0.01) and ML (*p* < 0.01) direction (shown in Figure 4B).

### Comparison of the number of muscle synergies in infant crawling

**Figure 5:**
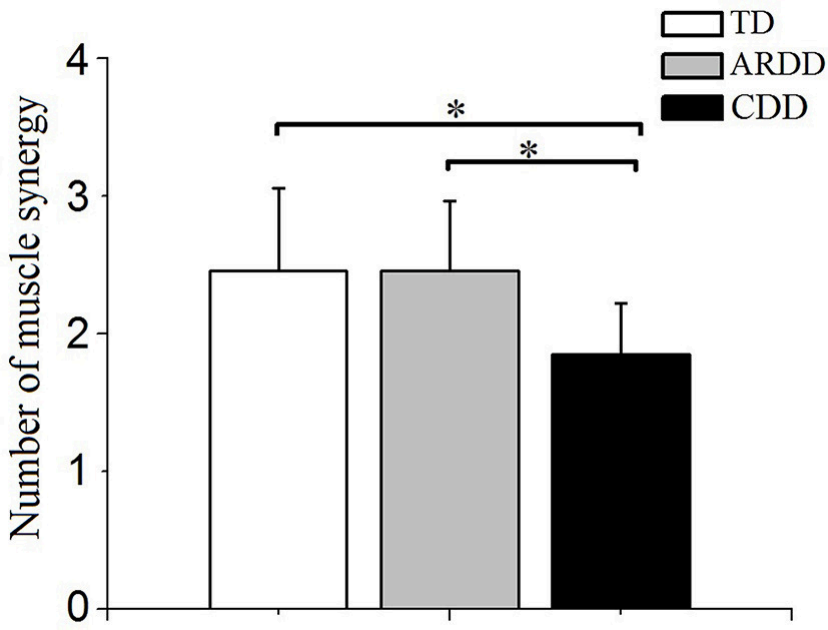
Comparison of the number of muscle synergies extracted with crawling data from typical developing infants (TD), infants at risk of developmental delay (ARDD), and infants with confirmed developmental delay (CDD). *indicates *p* < 0.05.

Of the 20 infants with TD measured, two synergies were identified in 60% (12 subjects), three synergies in 35% (7 subjects), and four synergies in 5% (1 subject). Of the 33 infants with ARDD measured, 45.5% (15 subjects) demonstrated two synergies, and 54.5% (18 subjects) three synergies. Of the 13 infants with CDD measured, 15.38% (2 subjects) demonstrated only one synergy and 84.62% (11 subjects) showed two synergies. There was a significant effect of group in the number of muscle synergies (*F* = 7.194, *p* = 0.002, observed power = 0.923) during crawling. A significantly reduced number of synergies (1.846 ± 0.375) was identified in infants with CDD during crawling compared with infants with TD (2.450 ± 0.604) and ARDD (2.450 ± 0.503), respectively. No significant differences were identified in the number of synergies observed between infants with TD and ARDD (Figure 5).

### Comparison of the number of co-activating muscles per synergy

**Figure 6:**
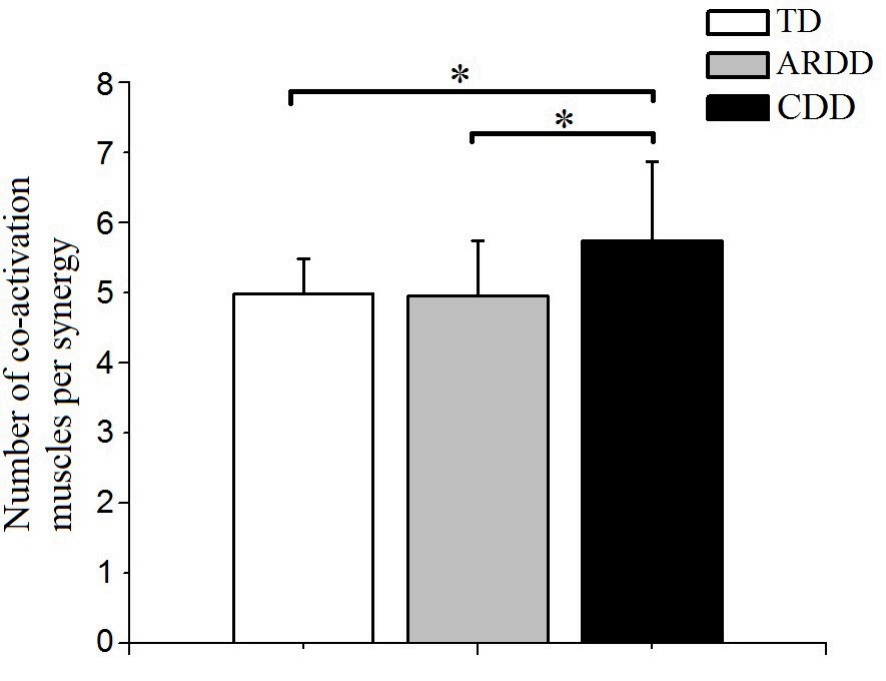
There was a significant difference in the number of co-activating muscles per synergy between typical developing infants (TD), infants at risk of developmental delay (ARDD), and infants with confirmed developmental delay (CDD). *indicates *p* < 0.05.

There was a significant effect of group in the number of co-activating muscles per synergy (*F* = 4.889, *p* = 0.011, observed power = 0.786). As shown in Figure 6, co-activation levels were significantly higher in the CDD group (5.730 ± 1.129) compared to the TD (4.979 ± 0.501, *p* = 0.03) and ARDD (4.95 ± 0.787, *p* = 0.012) group, respectively. There was no significant difference between TD and ARDD group (*p* > 0.05).

### Correlations of muscle synergy and kinematic indices

There were no significant correlations found between the number of co-activating muscles per synergy and crawling velocity, crawling cadence or normalized stance phase time.

**Figure 7:**
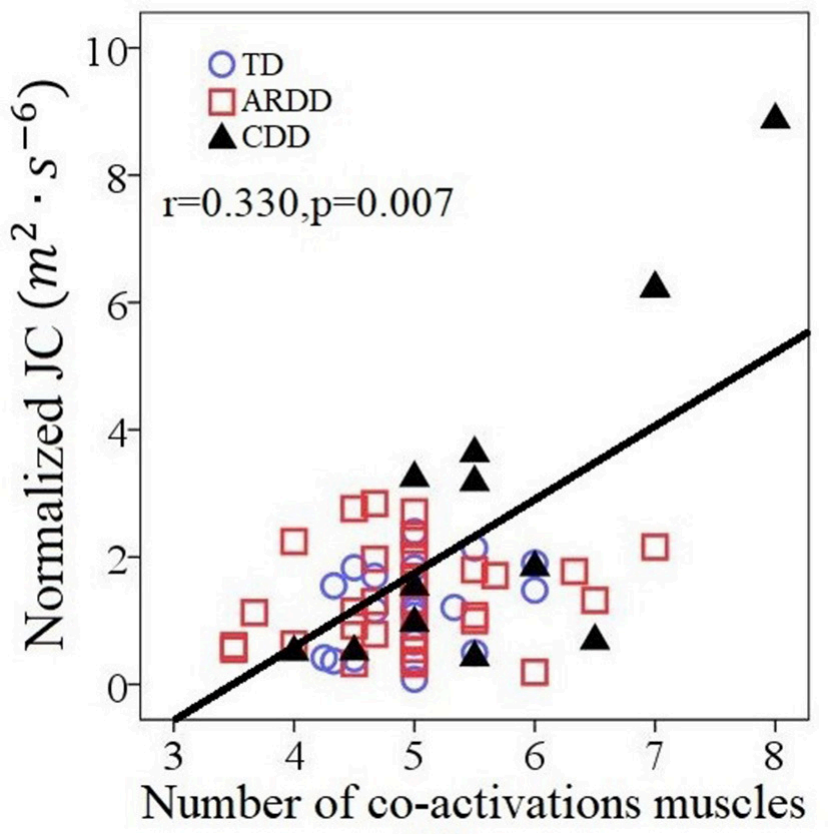
Correlations between the number of co-activating muscles per synergy and the normalized JC values for all 66 infants including typical developing infants (TD), infants at risk of developmental delay (ARDD), and infants with confirmed developmental delay (CDD).

Figure 7 reports the number of co-activating muscles per synergy plotted vs. normalized JC value. The number of co-activating muscles per synergy was significantly correlated (*r* = 0.330, *p* = 0.007) to the normalized JC value for all infants (Figure 7).

## Discussions

### Reduced movement smoothness during crawling in infants with developmental delay

During crawling on hands and knees, there was less smoothness in the movements of infants with known developmental delay. A prevailing hypothesis is that the central nervous system (CNS) is organized so that motor output strategy minimizes critical parameters of trajectory such as jerk (19) in order to achieve an accurate and smooth movement. This hypothesis has been supported by observations that those with neurological diseases affecting their CNS, such as stroke (20) and cerebral palsy (21), have less smoothness than control participants. Extending to the current study, our results of decreased smoothness in infants with developmental delay could be the result of a suboptimal motor command, due to the delayed or impaired development of the CNS (22). On the other hand, the main role of the CNS in generating smooth trajectories has been questioned by other authors (23) who suggested that the intrinsic properties of muscle tissue may be sufficient to produce smooth motion. Considering this hypothesis, the results of decreased smoothness could be related to muscle property differences, such those reported after neurological injury (24). Our results indicate that reduced smoothness of movement in CDD group could also emerge as a result of increased muscle co-activations, which is supported by the significant correlations between the number of co-activating muscles and the normalized JC value (Figure 7).

With regards the temporal-spatial parameters, the lack of significant difference between groups was likely because the recruited infants from the 3 groups are at a similar developmental age, as indicated by the clinical assessments.

### Constrained neuromuscular control strategy during crawling in infants with developmental delay

The number of muscle synergies identified in infants with CDD was lower compared to infants with TD and ARDD (Figure 5). The reduced number of muscle synergies were consistent with the results during walking in individuals with cerebral palsy (8), Parkinson's disease (25), and stroke (6), suggesting impaired muscle coordination. Our results suggest that infants with developmental delay, who were at high risk of cerebral palsy, have less degrees-of-freedom when coordinating muscles and show a constrained neuromuscular control. It is hypothesized that muscle synergies may represent a library of motor subtasks, and the CNS can flexibly combine them to produce complex and natural movements (26). Damage to the CNS, such as in cerebral palsy or stroke, disrupts this combination process, resulting in recruiting less subtasks (synergies). This points to the importance of intact descending control to appropriately recruit a full library of muscle synergies.

Our study also showed that the number of co-activating muscles per synergy was higher in infants with CDD compared to infants with TD and ARDD (Figure 6), which implies more muscle co-contractions during crawling in infants with developmental delay. Increased co-contraction of muscles was also shown in individuals with spinal cord injury (7), cerebral palsy and stroke during locomotion (27, 28). Previous studies have shown that a reduction in the descending signals resulted in higher co-activation of muscle (29). Therefore, higher muscles co-activations could indicate that developmental delay in the CDD group was the result of a brain injury in the infant's early life, even if it was not apparent from brain imaging.

### Clinical implications

Our results demonstrated that muscle synergy indices (such as the number of synergies and co-activating muscles per synergy) and kinematic output (normalized JC value) were significantly different between infants with confirmed developmental delay (CDD) and typical developing infants (TD), whereas these same variables did not show a significant difference between typical developing infants (TD) and infants at risk of development delay (ARDD). This result validates the concordance between metrics derived in this study and the clinical indicators of motor delay (i.e., Gesell Developmental Scale), suggesting that synergy indices and kinematic output variables are linked in a meaningful way with developmental delay on a group level. However, the group level for these more sensitive metrics can be extended to further understanding of individual participants, especially those at risk for developmental delay later in life.

These results of muscle synergy and movement smoothness analysis during crawling imply a different control strategy between infants with different risks or severity of developmental delay. In spite of their risk factors, the ARDD group was not found to be clinically delayed in the less sensitive clinical measures, but the presence of CNS impairment could become apparent in more sensitive metrics such as muscle synergy assessment, which may be useful in future for the development of more CNS specific rehabilitation plans.

### Limitations and opportunities for future work

This study quantified and compared the inter-limb muscle synergy and kinematics during crawling between typically developing infants and infants with developmental delay in a novel way. There are a few limitations that need to be acknowledged. Quadrupedal locomotion requires the coordinated behavior of many muscles of the arms, legs, and trunk (30). Because of the small size of infant's limb and the difficulty of measuring locomotion in infants, we measured four primary muscles from each of the arms and legs. In future work, the experimental protocol will be improved by measuring more skeletal muscles such as the gluteus maximus, abdominal and back muscles.

The cohorts of this study were matched on *developmental* age in order to collect data when they all had similar functional control over their body in order to successfully perform hands-and-knees crawling. However, due to motor delays there was a significant difference in the *chronological* age of the infant groups studied where the CDD group was older. If the hypotheses being tested were relative to the chronological or biological age of the CNS, the TD group could have been matched on chronologic age—but it would be anticipated that in that case an older cohort of TD children would have at least similar, if not better, skill in crawling than the current typical group, and thus may result even bigger difference between TD and CDD groups.

We also recognize that there are other potential confounding factors, such as different risk factors and etiologies for development delay (premature delivery, low birth weight, or lack of oxygen during birth), which may indicate different mechanisms for development delay. However, the investigation of these confounding factors is beyond the interests of the current study.

There are the two extreme data points in Figure 7, showing 2 CDD participants who demonstrated both very high JC values and co-activation of all or nearly all of the recorded muscles during crawling, which was visually different than the cluster formed by the TD group. One of them was identified by SPSS boxplot (use a step of 1.5 × interquartile range) as an “out” value. If we exclude this data point and the correlation within the rest 65 infants was still significant (*r* = 0.298, *p* = 0.016).). These two extreme data points suggests that they always activate all the muscles and lack the movement smoothness. Some of the infants with confirmed development delay (CDD) will likely receive a diagnosis of cerebral palsy, which is characterized with by the presence of spasticity and decreased selective motor control (31, 32). A future study should include follow-up with the CDD and ARDD infants, in order to ascertain if features of the crawling data are predictive of a later diagnosis of cerebral palsy.

Even considering the limitations above, the potential for assessing motor function and understanding of the state of the neuromuscular system during crawling period is an exciting prospect. Assessment of pathological impairment in motor control during walking can be conducted by gait analysis, which is been widely used in clinics and typically provides quantified metrics of kinematics and muscle activity (33). For those infants without walking ability, movement abnormalities are typically assessed by screening tests or visual analysis of their movement quality. This study demonstrates that utilizing more quantitative metrics can reveal impaired neuromuscular strategies before the onset of walking skills and provide insight for development of rehabilitation of protocols during infants' crawling stage. The long-term goal of this work is to develop a standardized measure, similar to gait analysis, that can assess motor function in infant crawling on an individual basis.

## Conclusion

This study demonstrated that infants with developmental delay demonstrated fewer inter-limb muscle synergies, increased number of muscles that co-contracted, and reduced movement smoothness during crawling on hands and knees, compared to typical developing infants. In addition, more co-activations across inter-limb muscles are considered to be attributable to the reduced movement smoothness in infant crawling. These muscle coordination and kinematic output deficits revealed impaired neuromuscular strategies during the infant crawling stage.

## Author contributions

WH, XW, NX, LC, and XZ designed the work. QX and YL collected the data. QX analyzed the data. JY, TS-M, and WH interpreted the data. QX and JY drafted the manuscript. TS-M and WH helped to create the final report.

### Conflict of interest statement

The authors declare that the research was conducted in the absence of any commercial or financial relationships that could be construed as a potential conflict of interest.
